# Performance of Kalon herpes simplex virus 2 assay using dried blood spots among young women in Uganda

**DOI:** 10.4102/ajlm.v5i1.429

**Published:** 2016-08-16

**Authors:** Sam L. Nsobya, Paul C. Hewett, Sam Kalibala, Barbara S. Mensch

**Affiliations:** 1 Department of Pathology, School of Biomedical Science, Makerere University, Kampala, Uganda; 2 Population Council, Washington, District of Columbia, United States; 3 Population Council, New York, New York, United States

## Abstract

This study evaluated the performance of the Kalon Biological HSV2 IgG enzyme-linked immunosorbent assay (Kalon Biological Ltd, Surrey, United Kingdom) on dried blood spots (DBS) of various dilutions compared with plasma from young women aged 18–24 years in Uganda. We estimated the sensitivity and specificity of three DBS dilutions using plasma as the reference. All three evaluated DBS dilutions yielded low sensitivities and specificities, with DBS 1:2 yielding the highest concurrence. Other herpes simplex virus type 2 assays should be examined with regard to their utility for testing DBS.

## Introduction

Herpes simplex virus type 2 (HSV-2) causes lifelong infections in exposed individuals and is a useful cumulative marker for unprotected sex. HSV-2 infection has also been shown to be an important co-infection for HIV, facilitating HIV acquisition and transmission, and accelerating disease progression.^1^ In Uganda, it is estimated that about half of adults aged 15–59 years are infected with HSV-2.^2^

Numerous studies have outlined the usefulness of dried blood spots (DBS) for the serologic diagnosis of infectious diseases, as well as for large-scale seroprevalence studies.^3,4,5,6,7^ Since DBS do not require immediate refrigeration, occupy little space and are easily transported, they are an attractive means for biomarker-based studies, particularly in geographic settings with limited laboratory resources.

Recently-developed type-specific HSV antibody tests are based on the detection of antibodies to glycoprotein G1 (gG1), a marker for HSV-1 infection, and glycoprotein G2 (gG2), a marker for HSV-2 infection.^8,9^ Validation of DBS for immunoglobulin G (IgG)-based tests has been conducted using HSV-1-positive antibody samples from 22 healthy volunteers.^10^ Hogrefe, Ernst and Su reported that testing data using a single dilution of DBS eluates (1:4) with the HSV type-specific enzyme-linked immunosorbent assay (ELISA) method were similar to those of sera diluted to 1:101 using the standard HerpeSelect^®^ ELISA (Focus Diagnostics, California, United States).^10^ The efficiency of using IgG eluted from DBS samples was found to be consistent with measurements of IgG concentrations in most corresponding serum samples.

However, results have been inconsistent when using the Kalon Biological HSV2 IgG ELISA assay (Kalon Biological Ltd, Surrey, United Kingdom; hereafter Kalon ELISA HSV2 assay) when measuring antibodies to gG2 with the same testing protocol.^10^ In a household survey conducted in 2010, sexual behavior data and DBS specimens were collected from young women aged 18–24 years in Kampala.^11^ When a 1:4 dilution was applied to our first 277 DBS specimens, the estimated HSV-2 prevalence was 2%. This prevalence was significantly lower than that reported previously in the 2004–2005 Uganda HIV/AIDS Sero-Behavioural Survey (UHSBS), which found a prevalence of 21% among women aged 15–19 years and 38% among women aged 20–24 years.^2^ When an additional 10 DBS specimens from our sample of young women were further analysed using a dilution of 1:2, the prevalence of HSV-2 increased three-fold.^11^

In the study reported here, we examined the performance of the Kalon ELISA HSV2 assay using DBS and plasma samples from stored specimens previously collected during the 2004–2005 UHSBS. The DBS laboratory assessment was conducted in late 2010 and early 2011. The study goal was to examine whether HSV-2 testing results based on DBS at various dilution levels were concordant with plasma-based results obtained from the same participants.

## Methods

### Ethical considerations

Participants who provided specimens to the 2004–2005 UHSBS consented to the long-term storage and future testing of their delinked blood specimens for which they would not receive results. We obtained additional permission for this study from the Uganda Ministry of Health. The study was also reviewed and approved by the Uganda Virus Research Institute’s Institutional Review Board (GC/127/11/10/15) and the Population Council Institutional Review Board (protocol 433).

### Study population

This study was conducted using existing stored DBS and plasma specimens from the 2004–2005 UHSBS. The UHSBS was a nationally-representative household-based survey that sampled 19 656 adult respondents. The main objective of the UHSBS was to obtain national and sub-national estimates of HIV prevalence and selected indicators of HIV-related risk behaviours, programme coverage and HIV knowledge and attitudes. One of its specific objectives was to determine the magnitude and distribution of HIV and other sexually-transmitted infections, such as HSV-2, in Uganda.^2^ The UHSBS estimated the national adult prevalence of HIV at 6.4% and that of HSV-2 at 44%.^2^

### Survey specimens and testing

Survey participants had venous blood samples drawn into 4.5 ml EDTA Vacutainer tubes, from which DBS were produced using Whatman SS903 specimen collection paper, air-dried overnight in plastic boxes and stored in lots of 20 separated by glassine paper in Ziploc bags containing desiccants. In the field, blood was centrifuged and the plasma was transferred to microvials. Plasma and DBS were transported periodically to a central laboratory in Entebbe for processing and storage at -80 °C (plasma) and -20 °C (DBS). The plasma and DBS samples for the UHSBS were tested for HSV-2 antibodies using the Kalon ELISA HSV2 assay within several weeks of collection. Results were classified as positive or negative using cutoffs as specified by the manufacturer.

### DBS validation study design

For the purposes of this study, we randomly selected 110 stored DBS specimens from women aged 18–24 years whose plasma-based equivalents tested positive and 110 stored DBS specimens whose plasma-based equivalents tested negative using the Kalon ELISA HSV2 assay. Laboratory testing and analysis was conducted at the Molecular Research Laboratory in Kampala as part of the Makerere University-University of California, San Francisco Research Collaboration on 2 May 2011.

From each of these 220 DBS, a 6 mm-diameter (28 mm^2^) disk, containing approximately 50–75 µL of blood per spot, was punched out from the filter paper and soaked overnight at 4 °C in 150 µL of phosphate-buffered saline (pH 7.4). After the overnight elution step, the eluates were diluted at three different levels, 1:4, 1:3 and 1:2. Each specimen was tested in triplicate with the Kalon ELISA HSV2 assay, as directed by the manufacturer’s instructions.

### Data analysis

Frequencies of reactivity, non-reactivity, sensitivity and specificity with 95% confidence intervals were generated using SAS^®^ software, version 9.3 (SAS Institute Inc., Cary, North Carolina, United States, 2011). DBS-based estimates of sensitivity and specificity were obtained using the plasma-based results as the reference. We determined the dilution level for DBS-based HSV-2 testing that yielded the highest (relative) sensitivity and specificity.

## Results

The final sample size included 210 individuals due to missing test results for 10 DBS. Of the 210 plasma specimens, 104 (49.5%) were reactive and 106 (50.5%) were non-reactive for HSV-2 antibodies (Table 1). The DBS 1:2 dilution yielded 116 (55.2%) reactive and 94 (44.8%) non-reactive results. DBS 1:3 produced 124 (59.0%) reactive and 86 (41.0%) non-reactive results, whereas DBS 1:4 yielded 110 (52.4%) reactive and 100 (47.6%) non-reactive results.

**TABLE 1:** HSV-2 seropositivity for plasma-based and DBS dilution-based assays using the Kalon ELISA HSV2 assay among young women in Uganda, 2004–2005.†

Plasma	DBS 1:2			DBS 1:3			DBS 1:4		
		
	Non-reactive‡	Reactive§	Total *n* (%)	Non-reactive‡	Reactive§	Total *n* (%)	Non-reactive‡	Reactive§	Total *n* (%)
Negative¶	78	28	106 (50.5)	68	38	106 (50.5)	72	34	106 (50.5)
Positive††	16	88	104 (49.5)	18	86	104 (49.5)	28	76	104 (49.5)

**Total *n* (%)**	**94 (44.8)**	**116 (55.2)**	**210 (100.0)**	**86 (41.0)**	**124 (59.0)**	**210 (100.0)**	**100 (47.6)**	**110 (52.4)**	**210 (100.0)**

DBS, dried blood spots; HSV-2, herpes simplex virus 2.

† Women were aged 18–24 years, *N* = 210;

‡ Non-reactive, DBS negative for HSV-2 antibodies;

§ Reactive, DBS positive for HSV-2 antibodies;

¶ Negative, plasma negative for HSV-2 antibodies;

†† Positive, plasma positive for HSV-2 antibodies.

The 1:2 ratio of buffer and eluate yielded the highest sensitivity (84.6%) and specificity (73.6%) (Table 2). The 1:3 dilution had the next-highest sensitivity (82.7%), but had the lowest specificity (64.2%). The 1:4 dilution showed the lowest sensitivity (73.1%) and a specificity of 67.9%. Overall, 127 (60.5%) of the 210 specimens had concordant results between plasma and all three DBS dilutions (not shown in the tables). Of the 127 concordant results, 69 (54.3%) were concordant positive and 58 (45.7%) were concordant negative. A total of 83 (39.5%) cases had a discordant result between plasma and at least one of the DBS dilutions. Of the 83 discordant results, 35 (42.2%) were discordant positive and 48 (57.8%) were discordant negative.

**TABLE 2:** Relative sensitivity, specificity and 95% confidence intervals of dried bloodspot dilutions tested with the Kalon ELISA HSV2 assay compared with plasma among young women in Uganda, 2004–2005.†

Dilution	Sensitivity % (95% CI)	Specificity % (95% CI)	Overall concordance %
DBS 1:2	84.6 (76.2–90.9)	73.6 (64.1–81.7)	79.0
DBS 1:3	82.7 (74.0–89.4)	64.2 (54.3–73.2)	73.3
DBS 1:4	73.1 (63.5–81.3)	67.9 (58.2–76.7)	70.5

DBS, dried blood spots.

† Women were aged 18–24 years, *N* = 210.

## Discussion

The Kalon ELISA HSV2 assay has been shown to be sensitive and specific for HSV-2 diagnosis using plasma.^2,12^ In our study, a dilution of 1:2 showed the highest sensitivity and specificity compared with the 1:3 and 1:4 dilution levels. This estimated sensitivity and specificity is low relative to the plasma-based reference results. Specifically, our findings differ from the estimated higher sensitivity and specificity found in a South African population which used the HerpeSelect ELISA serological assay.^13^ However, earlier studies evaluating the plasma-based HerpeSelect ELISA test in sub-Saharan populations suggested a lower specificity than the plasma-based Kalon ELISA HSV2 assay.^14^

In addition, patterns of reactivity varied by dilution level. Although not the optimal dilution based on sensitivity or specificity, DBS 1:3 had the highest proportional reactivity to HSV-2 antibodies (59.0%). All three dilutions yielded higher frequencies of reactivity than plasma (49.5%), specifically DBS 1:2 (55.2%), DBS 1:3 (59.0%) and DBS 1:4 (52.4%). We found low concordance between plasma-based and DBS-based results, which contrasts with the high concordance reported by Hogrefe et al. using the HerpeSelect assay.^10^

### Limitations

There are several limitations in our study. The sampling frame was limited to young women aged 18–24 years in Uganda; thus, our results are not generalisable to men or older adults. Additionally, the sample size was relatively small. Further, we did not compare the quantity of IgG in the DBS punch specimens to that in plasma specimens. Finally, this study only used the Kalon ELISA HSV2 assay to evaluate DBS for HSV-2 diagnosis. Other HSV-2 assays, such as the HerpeSelect 2 ELISA IgG and Biokit HSV2 Rapid Assay are used in African countries and need to be further evaluated to compare their utility with DBS.

### Conclusion

In summary, our study examined the performance of the Kalon ELISA HSV2 assay, using DBS in a population of young women in Uganda. DBS testing with the Kalon ELISA HSV2 assay revealed relatively low sensitivity and specificity compared with plasma-based results on the same individuals. While DBS would be an appealing and relatively simple means for biomarker-based studies, particularly in resource-constrained settings, the accuracy of this testing format would need to be substantially improved before its use could be recommended for epidemiological studies.
